# Cervical Dysplasia and Treatments Barrier in Jail: A Study in Marseille's Detention Center—Les Baumettes, France

**DOI:** 10.1089/whr.2021.0135

**Published:** 2022-08-04

**Authors:** Claire Delage de Luget, Camille Jauffret, Cindy Faust, Sophie Knight, Christophe Bartoli, Emilie Ricard

**Affiliations:** ^1^ Department of Obstetrics and Gynecology, Nord Hospital, Assistance Publique-Hopitaux de Marseille, Chemin des Bourrely, Marseille, France.; ^2^ Department of Surgical Oncology, Paoli Calmettes Institute, Marseille, France.; ^3^ EA3279, CEReSS, Health Service Research and Quality of Life Center, Aix-Marseille University, Marseille, France.; ^4^ Department of Forensic Pathology, Assistance Publique-Hôpitaux de Marseille (APHM), CHU Timone, Marseille Cedex, France.; ^5^ Department of Obstetrics and Gynecology, Salon de Provence Hospital, France.

**Keywords:** cervical dysplasia, cervical cancer screening, correctional facility, prison, cervical cancer, quality of life

## Abstract

**Introduction:**

The main objective was to estimate the prevalence of cervical dysplasia among incarcerated women. The secondary objective was to identify obstacles to the possible management of a cervical dysplasia in detention by assessing their knowledge of screening for cervical cancer (CC), the existence of vaccination, and the management of precancerous lesions.

**Materials and Methods:**

The first part of the study was descriptive and retrospective, studying pap-smear results in women's correctional facility at the Baumettes prison center (PC) in Marseille, France. The second part of the study was qualitative and prospective and took place at the Baumettes PC. Voluntary and French-speaking inmates aged 25–65 years answered an short-form 12 quality-of-life questionnaire and a more targeted questionnaire on CC screening and cervical dysplasia treatments.

**Results:**

In total, 201 pap-smear tests were assessed, 135 were normal (66.8%) and 33 unsatisfactory (16.3%). There were 33 abnormal pap-smear tests (16%). The patients were 38.9 years (±9.5 years), had 4.05 pregnancies (±2.7), and 2.29 children (±1.85). Seventy-five percent were smokers. Psychiatric disorders were found in 52.2% inmates. In the second part of the study, among the 35 inmates questioned, the SF-12 questionnaire's analysis shows that the physical health component score was on average 43.6 and the mental health component score (MCS) was 36.5. Analysis demonstrated that the uncertainty of the exact day of hospitalization is an obstacle to treatment for 15 patients presenting significantly a lower MCS score (*p* = 0.047).

**Conclusion:**

Prevalence of pathological pap-smear tests is higher within a prison population, screening is accepted and the inmates are receptive to information about CC prevention, delivered during individual interviews. Mental health's management and care system's reorganization in detention are essentials factors for care acceptance.

## Introduction

Cervical cancer (CC) is a preventable cancer. Screening has reduced the incidence of this cancer in developed countries. In France, in 2020, 3379 new cases of CC were identified, and 1452 women died.^1^ It is a viral induced cancer in 98.2% of cases by *human papillomavirus* (HPV) persistent infection.^2^ Since July 2019, CC screening is organized and based on pap-smear and HPV test.^3^ CC is a slow-growing cancer characterized by curable precancerous lesions. It can be detected at an early stage and be prevented by dysplasia detection.^4^

Certain categories of women are at higher risk of developing CC.^5^ In the 1960s, American literature showed that prevalence of CC was higher among female imates.^6–8^ This can be explained by the fact that risk factors of CC are more frequent in this population^9^: HIV infection, tobacco consumption, and opioid use. These women, before their detention, are part of a population that has a poor access to the health care system.^10^

The main objective was to estimate the prevalence of cervical dysplasia in incarcerated women. The secondary objective was to identify obstacles to the possible management of a cervical lesion in detention by assessing the knowledge of inmates on screening of CC, on the existence of vaccination, and on the management of precancerous lesions.

## Materials and Methods

In France, since January 1994, the public service guarantees health in prison. Prevention and care in detention are ensured by functional units within the detention centers.

Our study was organized into two parts. It took place at the Baumettes prison center in Marseille, which includes a *maison d'arrêt* and a detention center. On September 1, 2021, in France, 2041 women were detained, 154 (7.5%) were inmates at the Baumettes.

### First part

The first part of the study was descriptive, and retrospective, based on medical files studying all pap-smear tests performed between April 21, 2015, and June 5, 2019, at the Baumettes prison in Marseille for inmates aged 25 to 65 years.

### Data

Clinical data were extracted from medical records. For each patient, the following information was collected: age, family status, number of children, educational level, profession, and nationality. We also collected information on addictions: tobacco consumption, cannabis, and intravenous and/or nasal drug use.

The following medical data were collected: existence of a psychiatric disorder, means of contraception, history of abortion, menopausal status, presence of sexually transmitted diseases, and seropositivity for HIV.

The judicial status (accused or convicted detainee) was collected. Finally, we extracted information on the screening and treatment of cervical lesions: pap-smear test, HPV test, realization of a colposcopy and its result, realization of a conization, and its result.

### Second part

The second part of the study was qualitative and prospective. Inmates of the Baumettes detained center, volunteers, and French speaking answered a questionnaire (Supplementary Appendix SA1). It was a quality-of-life questionnaire composed of the short-form 12 (SF-12) and a more targeted questionnaire on CC screening, written by the authors.

The SF-12 questionnaire is a general health assessment questionnaire: it combines synthetic information (12 items) that combines a score on the physical dimension physical health component score (PCS) and a score on the mental dimension mental health component score (MCS).^11^ This questionnaire has been validated in the literature with the jail population.^12–14^

### Study population

The inclusion criteria in the study were as follows:

Inmates aged 25 to 65 years at the time of the study
Volunteers
Good understanding of the French language.

### Collection methods

The patients were received for an individual interview during which they filled out the questionnaire in the presence of the principal investigator.

### Ethics

The retrospective part of the study was registered in the register of treatment activities of the Assistance Publique-Hôpitaux de Marseille, in accordance with the General Data Protection Regulations under the number Portail d'Accès aux données de santé: 21-225.

The prospective part of the study received a favorable opinion from the ethics committee of the faculty of medicine of the University of Aix-Marseille, France.

### Statistical analysis

For the first part of the study, all the data were collected on an anonymized database using a spreadsheet on Microsoft Excel 2021^®^.

To meet the main objective of this study, a descriptive analysis of the data was performed using R© software version 3.6.1. Quantitative variables are presented by means, and standard deviations, qualitative variables as counts and percentages. Two groups were compared (normal smear and pathological smear) in multivariate analysis using a logistic regression model. The qualitative variables were compared with the Fisher test. The differences between the quantitative variables were analyzed with Student's *t*-test.

For the second part of the study, a descriptive analysis of the data was also carried out using R software version 3.6.1.

The secondary endpoint was defined and sought by the following two variables: “uncertainty about the exact day of hospitalization” and “deprivation of activity” (Supplementary Appendix SA1).

The results of the SF-12 questionnaire were interpreted by a computer algorithm.

Mean comparisons (analysis of variance [ANOVA]) and logistic regression models were performed using IBM SPSS© 26.0 software.

All analyses were performed with a risk of species 1*α* = 5%, a *p*-value <0.05 was considered statistically significant.

## Results

### Retrospective study

#### Descriptive statistics

From April 2015 to June 2019, 214 pap-smear tests were performed. Eleven were not included in our database because the patients were <25 years old, and two patients were excluded from the database because their medical files were lost.

Patients characteristics were as follows (Table 1): mean age was 38.9 years old (±9.5 years), an average of 4.05 pregnancies (±2.7), of 2.29 children (±1.85), and underwent an abortion in average 1.22 times (±1.51). Most of the inmates were French (132, or 65%), 50.2% of them were convicted (102), and 34.5% were accused (70).

**Table 1.** Clinical Characteristics of Patients in the Retrospective Study

Average age in years (SD)	38.39 (9.5)
Average gesture (SD)	4.05 (2.7)
Average parity (SD)	2.29 (1.85)
Average no. of abortions (SD)	1.22 (1.51)
Smoking (%)	
Smoker	152 (75.6%)
Nonsmoker	41 (20.4%)
MD	8 (4%)
Cannabis use (%)	
Yes	46 (23%)
No	137 (68%)
MD	18 (9%)
Intravenous and/or nasal drug use (%)	
Yes	35 (17.4%)
No	147 (73.1%)
MD	19 (9.5%)
Psychiatric disorders (%)	
Yes	105 (52.2%)
No	84 (41.8%)
MD	12 (6%)
Initial gynecological consultation (%)	
Yes	166 (82.6%)
No	3 (1.5%)
MD	32 (15.9%)
HPV vaccination (%)	
Yes	3 (1.5%)
No	171 (85%)
MD	27 (13.4%)
Sexually transmitted disease (%)	
Yes	19 (9.5%)
No	160 (79.6%)
MD	22 (11%)
HIV positive (%)	
Yes	9 (45%)
No	168 (83.5%)
MD	24 (12%)
Method of contraception (%)	
Yes	53 (26.3%)
No	134 (66.7%)
MD	14 (7%)
Menopausal status (%)	
Menopaused	28 (14%)
Nonmenopaused	163 (81%)
MD	10 (5%)
Nationality (%)	
French	132 (65%)
Eastern Europe	24 (11.8%)
Sub-Saharan Africa	10 (4.9%)
North Africa	8 (3.9%)
Western Europe	6 (2.9%)
South America	3 (1.5%)
MD	19 (9.8%)
Family status (%)	
Single	83 (40%)
In a relationship	57 (28%)
MD	62 (31%)
Level of education (%)	
Baccalaureate±higher studies	9 (4.43%)
High school and professional certificates	39 (19.2%)
Middle school	36 (17.7%)
Out of school, primary school	6 (2.96%)
MD	113 (55.6%)
Professional activity (%)	
Yes	57 (28.4%)
No	71 (35.3%)
MD	73 (36.3%)
Judicial status (%)	
Detainee warned	70 (34.5%)
Sentenced prisoner	102 (50.2%)
MD	30 (15.3%)

HPV, human papillomavirus; MD, missed data; SD, standard deviation.

Forty percent of inmates were single at the time of their incarceration and 35% had no professional activity. Level of education was unknown for 113 patients, 39 (19.2%) went to high school or professional certificate, 36 (17.7%) to middle school, 9 (4.43%) had graduated, and 6 patients did not go to school or only went to primary school.

In their medical history, psychiatric disorders were found for 52.2% of them. Nineteen patients had had a sexually transmitted disease, and nine were HIV positive. Only three patients were vaccinated against HPV.

Seventy-five percent of patients were smokers, 23% of them consumed cannabis, and 17.4% used intravenous or inhaled drugs.

Finally, 53 patients had contraception (26.3%) and 28 were postmenopausal (14%).

Among the 201 pap-smear tests performed, 135 were normal (66.8%) and 33 unsatisfactory (16.3%). There were 33 abnormal pap-smear tests (16%). The distribution of abnormal pap-smears was as follows (Table 2): 11 concluded to atypical squamous cells of undetermined significance (ASC-US) lesions, 7 high-grade lesions, 6 atypical squamous cell evocating high-grade lesions , 6 low-grade lesions, and 3 atypical glandular cells lesions.

**Table 2.** Pap-Smear Results

Results	*N* = 201 (100%)
Normal	135 (66.8%)
Unsatisfactory	33 (16.3%)
Low-grade lesion	6 (3%)
High-grade lesion	7 (3.5%)
Atypical squamous cells of undetermined significance	11 (5.5%)
Atypical squamous cells evocating high-grade lesions	6 (3%)
Atypical glandular cells	3 (1.5%)

Among the 20 HPV tests performed after ASC-US lesions or as primary screening after 2019, 11 were found to be positive (5.4%) and 9 negative (4.4%).

As part of the management of abnormal smear results, 27 colposcopies were indicated (Table 3). One was not performed because the patient refused the examination. Colposcopic impressions and biopsy results were as follows: 12 high grade squamous intraepithelial lesion, 6 low grade squamous intraepithelial lesion, 1 *in situ* carcinoma was found, 2 were unsatisfactory; 4 colposcopies were found to be normal and 1 found metaplasia.

**Table 3.** Colposcopy Results

Results	*N* = 27 (100%), *n* (%)
Not performed	1 (4)
Metaplasy	1 (4)
In situ carcinoma	1 (4)
Low-grade lesion	6 (24)
High-grade lesion	12 (48)
Normal	4 (16)
Unsatisfactory	2 (8)

After colposcopies, eight conizations were indicated (Table 4). Two patients with high-grade lesions refused the operation. The anatomopathological results of the conizations performed concluded to two high-grade lesions, two high-grade lesions with pathological limits, one adenocarcinoma, and one squamous cell carcinoma.

**Table 4.** Conizations Results

Results	*N* = 8
Inmates refusal	2
Epidermoid carcinoma	1
Adenocarcinoma	1
In sano high-grade lesion	2
Not in sano high-grade lesion	2

### Subgroup analysis

By comparing prisoners with a normal pap-smear result (135) with those with an abnormal pap-smear result (33), no significant difference was found between the two groups even after adjusting for tobacco (*p* = 0, 08) and contraception (*p* = 0.11).

### Prospective study

#### Descriptive statistics

On June 25, 2021, 112 inmates were eligible for our study. From June to September 2021, an interview was offered to 48 patients, 4 had been released or transferred before the interview, 1 was hospitalized, and 8 refused the interview. A total of 35 patients answered the questionnaire.

The respondents (Table 5) were 40.86 years old (±11.6). Seventy-four percent of them (26) were smokers. Most inmates had children (32), and of these, 56% had at least 3 children.

**Table 5.** Clinical Characteristics of Patients in the Prospective Study

Average age in years (SD)	40.86 (11.6)
Smoking (%)	
Smoker	26 (74)
Nonsmoker	6 (17)
Weaned	3 (8)
Child (%)	
Yes	32 (91)
No	3 (9)
No. of children (%)	
1	8 (25)
2	6 (18)
3 and more	18 (56)

Eighteen inmates saw their time in detention as an opportunity for a health check-up, but 20 (57%) inmates feared that they would be poorly taken care of if they had a health issue during their incarceration.

Regarding CC screening (Table 6), 71.4% (25) of them were aware of the existence of organized screening and had had a pap-smear test in the past 3 years. Half of the inmates (48%) knew that CC was caused by HPV infection and that it was mainly sexually transmitted. However, 21 patients were not aware of the existence of HPV vaccination, and only 1 inmate reported having been vaccinated against HPV. After explanations, if 83% of inmates did not know the principle of conization, 30 (86%) of them considered the information provided sufficient and 25 (71%) reassuring.

**Table 6.** Short Form 12 Survey: Physical Health Component Score and Mental Health Component Score (Out of 100 Points)

	Average (SD)	Median (min–max)
PCS score	43.6 (11.4)	39.7 (21.6–62.3)
MCS score	36.5 (12.9)	32.6 (16.4–62.3)

MCS, mental health component score; PCS, physical health component score.

### Analysis of the SF-12 questionnaire

From the 12 questions, two scores were calculated (Table 7). The PCS score assessing the state of physical health was on average 43.6/100 (±11.4) in our group of inmates, corresponding to a slight disability. The MCS score evaluating the psychic component was, on average, 36.5/100 (±12.9), which corresponds to a moderate disability.

**Table 7.** Gynecological and Hospitalization-Related Surveys

Questions	*n* (%)
Do you see the time of your detention as an opportunity to take stock of your health?	
Yes	18 (51.4)
No	11 (31.4)
Never think about it	4 (11.4)
No opinion	2 (5.7)
As a prisoner, do you think that you are treated the same way by health professionals?	
Yes	18 (51.4)
No	12 (34.3)
No opinion	5 (14.3)
Do you know that even if you are in detention, you have the same health rights as the general population?	
Yes	31 (88)
No	4 (11.4)
Are you afraid of being poorly taken care of if you have a health problem while in detention?	
Yes	20 (57)
No	11 (31)
No opinion	4 (11.4)
Did you know of the existence of this screening?	
Yes	25 (71.4)
No	10 (28.5)
Have you had a smear or HPV-test in the past 3 years?	
Yes	25 (71.4)
No	9 (25.7)
Do not remember	1 (2.8)
CC is mainly caused by infection with a virus: the human papilloma virus, did you know?	
Yes	17 (48)
No	18 (51)
This is a virus whose transmission is predominantly sexual, did you know that?	
Yes	17 (48)
No	18 (51)
There is a vaccine to protect against this virus, did you know that?	
Yes	14 (40)
No	21 (60)
Have you been vaccinated against CC?	
Yes	1 (3)
No	29 (83)
Do not remember	5 (14)
Did you know the principle of colposcopy?	
Yes	12 (34)
No	23 (66)
Have you ever had one?	
Yes	5 (14)
No	30 (85)
Did you know about conization and its principles?	
Yes	6 (17)
No	29 (83)
Do the explanations seem sufficient to you?	
Yes	30 (86)
No	2 (6.5)
Do not know	3 (8.5)
Do the explanations seem reassuring to you?	
Yes	25 (71)
No	4 (11
Do not know	6 (17
Do you know the UHSI, the interregional secure hospital unit?	
Yes	25 (71)
No	10 (29)
Have you ever been hospitalized there?	
Yes	20 (43)
No	15 (57)
If yes: do you have a negative memory of your hospitalization?	
Yes	7 (41)
No	8 (59)
If not: do you have a negative image of this service?	
Yes	0
No	11 (55)
Never think about it	9 (45)
Do you think that being hospitalized at UHSI deprives you of certain activities that you usually have in detention?	
Yes	17 (48)
No	9 (25)
Do not know	9 (25)
Is it a drag if you had to go to this service?	
Yes	11 (31.4)
No	17 (48.6)
Do not know	7 (20)
Is the uncertainty of your exact day of hospitalization a barrier to going to UHSI?	
Yes	15 (43)
No	17 (48.5)
Do not know	3 (8)
Does having an outpatient operation seem more acceptable to you as a mode of hospitalization?	
Yes	20 (57)
No	7 (20)
Do not know	8 (23)

CC, cervical cancer; UHSI, unité hospitalière sécurisée interrégionale.

### Comparison of means tests (ANOVA)

After a comparison of means test, it is demonstrated that the uncertainty of the day of hospitalization is an obstacle to treatment for 15 patients, presenting significantly lower MCS scores (*p* = 0.047). Restricting activities during hospitalization is not an obstacle to management in these same patients (*p* = 0.69). These two variables do not appear to be barriers to treatment in patients with a lower PCS score (*p* = 0.88 and *p* = 0.4).

### Univariate and multivariate analyses

After univariate analysis (Table 8), the link between MCS score and refusal of treatment due to uncertainty about the date of hospitalization was at the limit of significance (*p* = 0.052). There is no significant link between the fear of being poorly taken care of if one presents a health issue in detention and the refusal of hospitalization due to the uncertainty of the exact day of hospitalization (*p* = 0.08). Multivariate analysis did not find any significant difference between the two groups (*p* = 0.15).

**Table 8.** Analysis of the Secondary Endpoint (Univariate Analysis)

	Uncertainty = no brake (*n* = 17)	Uncertainty = brake (*n* = 15)	Odds ratio	[Confidence interval]	*p*
PCS score average (SD)	43.24 ± 10.6	42.44 ± 11.83	0.993	[0.932–1.059]	0.834
MCS score average (SD)	41.67 ± 13.97	32.79 ± 8.91	0.938	[0.879–1.001]	0.052
Fear					
No (%)	8 (50)	2 (13.4)	1.00		
Yes (%)	9 (50)	10 (66.6)	5.00	[0.821–30.46]	
Do not know (%)	0 (0)	3 (20)			0.081

Fear = “Are you afraid of being poorly taken care of if you have a health problem while in detention?”

## Discussion

In our study, the prevalence of an abnormal pap-smear result is 16%.

Although there are no national data on cytological abnormalities in France, the latest study in Ile de France found 3% of abnormal smears,^15^ which suggests that there are up to five times more abnormal pap-smear tests in our inmate population than in the general population. In our study as in the literature, ASC-US results are the most represented.

Our inmate population presents the identified risk factors for CC (other than HPV infection): smoking,^16^ multiparity, sexually transmitted diseases, and low socioeconomic level. However, as in the publication by Binswanger et al.,^17^ our sample of inmates highly adhered to CC screening. Ramaswamy and Kelly^18^ show in their focus groups conducted in Kansas prisons that inmates are receptive to prevention and education on CC, which we also found in our interviews. Several authors^19,20^ show that after intervention with inmates, screening for CC at 1 and 3 years has improved.

Beyond screening, primary prevention of CC involves the anti-HPV vaccination. Vaccination began in France in September 2006 initially for young girls aged 14 years and over. Both in the database and in the interviews, it appears that a very small number of inmates are vaccinated against HPV. The literature does indeed identify poorer vaccination coverage in disadvantaged and less educated areas^21,22^ from which our population comes.

The secondary objective of our study was to identify the obstacles to the management of a cervical dysplasia in detention. Uncertainty about the exact day of hospitalization, as is the rule in detention, associated with a low MCS score is factors that significantly led to refusal of care. However, our study could not establish a statistically significant link between the refusal of care and the MCS score, probably because of its lack of power.

The inmates questioned consider that they have an altered state of physical and mental health, which is consistent with data from French^23,24^ and international^25–27^ literature and its improvement is a public health issue targeted by the Agence Régionale de Santé.^28^

Outpatient care could prove to be an alternative to hospitalization, acceptable for 20 of the 35 inmates in our study (Table 4), and thus limits the obstacles to care. The Besançon hospital even proposes conization in consultation, with an 88% satisfaction among the patients questioned.^29^

The strength of our study lies in the retrospective database that supports the results of the qualitative part. This is the first French study to focus on cervical lesions and their management in prison.

Our study has limitations. Since July 2019, screening for CC is organized and HPV test is used to screen women >30 years old with normal pap-smear test. Our database was conducted during a period before these changes, a low number of HPV tests were performed (11%), and it may not reflect the latest results obtained by the implementation of organized screening. In addition, access to colposcopy in the detention center has improved by the acquisition in 2018 of a colposcope.

We were only able to interview a small sample of inmates, but we chose to conduct individual interviews to ensure understanding and the quality of responses. In addition, the constraints due to prison system only allowed a small number of interviews during the study period.

## Conclusion

Our study shows that if the prevalence of pathological pap-smear tests is higher within a prison population, screening is accepted and the inmates are receptive to information about CC prevention, delivered during individual interviews. Mental health's management and care system's reorganization in detention are essential factors for care acceptance.

## Supplementary Material

Supplemental data

## Supplementary Material

Supplementary Appendix SA1

## Abbreviations Used

ANOVA: analysis of variance
ASC-US: atypical squamous cells of undetermined significance
CC: cervical cancer
HPV: human papillomavirus
MCS: mental health component score
MD: missed data
PC: prison center
PCS: physical health component score
SD: standard deviation
SF-12: short form 12
UHSI: unité hospitalière sécurisée interrégionale
