# Supplementary material for: Cervical Dysplasia and Treatments Barrier in Jail: A Study in Marseille's Detention Center—Les Baumettes, France
Source: Womens Health Rep (New Rochelle). 2022 Aug 4;3(1):670–7. doi: 10.1089/whr.2021.0135 (PMC9436383; doi:10.1089/whr.2021.0135)
Supplement: Supplemental data [file Suppl_AppSA1.docx]

# **Appendix**

Appendix 1. Questionnaires

**Première Partie : Questionnaire de Qualité de vie SF-12**

1 Dans l’ensemble, pensez-vous que votre santé est :

☐1 Excellente ☐2 Très bonne ☐3 Bonne ☐4 Médiocre ☐5 Mauvaise

En raison de votre état de santé actuel, êtes-vous limitée pour : 
2. des efforts physiques modérés (déplacer une table, passer l’aspirateur, jouer aux boules...) ?  
☐1 Oui, beaucoup limitée ☐2 Oui, un peu limitée ☐3 Non, pas du tout limitée   

3. monter plusieurs étages par l’escalier ?

☐1 Oui, beaucoup limitée ☐2 Oui, un peu limitée ☐3 Non, pas du tout limitée

Au cours de ces 4 dernières semaines, et en raison de votre état physique :

4. avez-vous accompli moins de choses que vous auriez souhaité ?

☐1 Oui ☐2 Non

5. avez-vous été limitée pour faire certaines choses ?

☐1 Oui ☐2 Non

Au cours de ces 4 dernières semaines, et en raison de votre état émotionnel (comme vous sentir triste, nerveuse ou déprimée) :

6. avez-vous accompli moins de choses que vous auriez souhaité ?

☐1 Oui ☐2 Non

7. avez-vous eu des difficultés à faire ce que vous aviez à faire avec autant de soin et d’attention que d’habitude ?

☐1 Oui ☐2 Non

8. Au cours de ces 4 dernières semaines, dans quelle mesure vos douleurs physiques vous ont-elles limité dans votre travail ou vos activités domestiques ?

☐1 Pas du tout ☐2 Un petit peu ☐3 Moyennement ☐4 Beaucoup ☐5 Énormément

Les questions qui suivent portent sur comment vous vous êtes sentie au cours de ces 4 dernières semaines. Pour chaque question, indiquez la réponse qui vous semble la plus appropriée.

|  | Toujours | La plupart du temps | Souvent | Parfois | Jamais |
| --- | --- | --- | --- | --- | --- |
| 9.Y a-t-il eu des moments où vous vous êtes sentie calme et détendue ? |  |  |  |  |  |
| 10.y a-t-il eu des moments où vous vous êtes sentie débordante d’énergie ? |  |  |  |  |  |
| 11.y a-t-il eu des moments où vous vous êtes sentie triste et abattue ? |  |  |  |  |  |

12. Au cours de ces 4 dernières semaines, y a-t-il eu des moments où votre état de santé physique ou émotionnel vous a gêné dans votre vie sociale et vos relations avec les autres, votre famille, vos amis, vos connaissances ?  

☐1 Toujours ☐2 La plupart du temps ☐3 Souvent ☐4 Parfois ☐5 Jamais

**Deuxième Partie :**

- Quel est votre âge ?
- Fumez-vous du tabac ?
  - Oui
  - Non
  - J’ai arrêté
- Avez-vous des enfants ?
  - Oui
  - Non
- Si oui, combien ?
  - 1
  - 2
  - 3 et plus
- Voyez-vous le temps de votre détention comme une occasion de faire un bilan sur votre état de santé ?
  - Oui
  - Non
  - Je n’y avais pas pensé
  - Je n’ai pas d’avis
- Pensez-vous, en tant que détenue, être traitée de la même manière que la population générale par les professionnels de santé ?
  - Oui
  - Non
  - Je ne sais pas
- Savez-vous que même si vous êtes en détention, vous disposez des mêmes droits à la santé que la population générale ?
  - Oui
  - Non
- Avez-vous peur d’être mal prise en charge si vous avez un problème de santé pendant votre détention ?
  - Oui
  - Non
  - Je ne sais pas

**Troisième Partie :**

Pour les femmes entre 25 et 29 ans, le test de dépistage est réalisé par frottis tous les 3 ans, après deux premiers tests réalisés à 1 an d’intervalle et dont les résultats sont normaux.

Pour les femmes de 30 ans à 65 ans, le test HPV-HR est réalisé 3 ans après le dernier frottis dont le résultat est normal. Un nouveau test est refait tous les 5 ans, jusqu’à l’âge de 65 ans, dès lors que le résultat du test est négatif.

Le frottis et le test HPV-HR se fait par un médecin ou une sage-femme lors d’un examen gynécologique, après la pose du spéculum, la cytobrosse balaie le col de l’utérus.


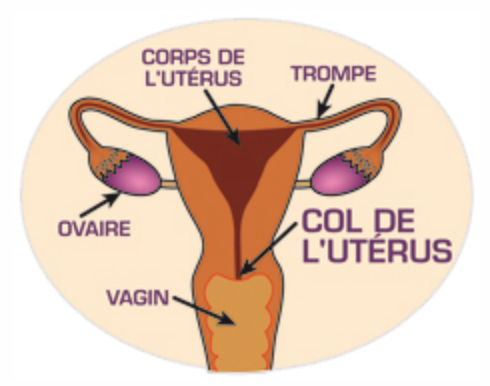

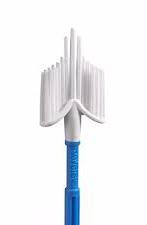
cytobrosse

- Connaissiez-vous l’existence de ce dépistage ?
  - Oui
  - Non
- Avez-vous eu un frottis ou un test HPV-HR dans les trois dernières années ?
  - Oui
  - Non
  - Je ne m’en souviens pas

Le cancer du col utérin est principalement dû à l’infection par un virus : l’human papilloma virus (HPV)

- Le saviez-vous ?
  - Oui
  - Non

Il s’agit d’un virus dont la transmission est essentiellement sexuelle.

- Le saviez-vous ?
  - Oui
  - Non

Cependant, il existe un vaccin pour se protéger de ce virus. En France, il est proposé gratuitement en 2 injections à toutes les jeunes filles entre 11 et 14 ans, et entre 14 et 19 ans si cela n’a pas été fait.

- Le saviez-vous ?
  - Oui
  - Non
- Avez-vous été vaccinée contre le cancer du col de l’utérus ?
  - Oui
  - Non
  - Je ne m’en souviens pas

Être vaccinée n’empêche pas de se protéger pendant les rapports sexuels et de faire le dépistage par frottis cervico-utérin.

Dans certains cas, le résultat du frottis est anormal et montre des lésions (anomalies) qu’il faut aller explorer par un examen appelé colposcopie.

C’est un examen indolore, qui se fait en position gynécologique.

Le colposcope est un microscope qui explore le col de l’utérus. Deux produits liquides sont appliqués sur le col pour identifier d’éventuelles lésions.

Si des lésions sont repérées, des biopsies (petits prélèvements) sont réalisées et envoyées en analyse. Ces biopsies ne sont pas douloureuses.


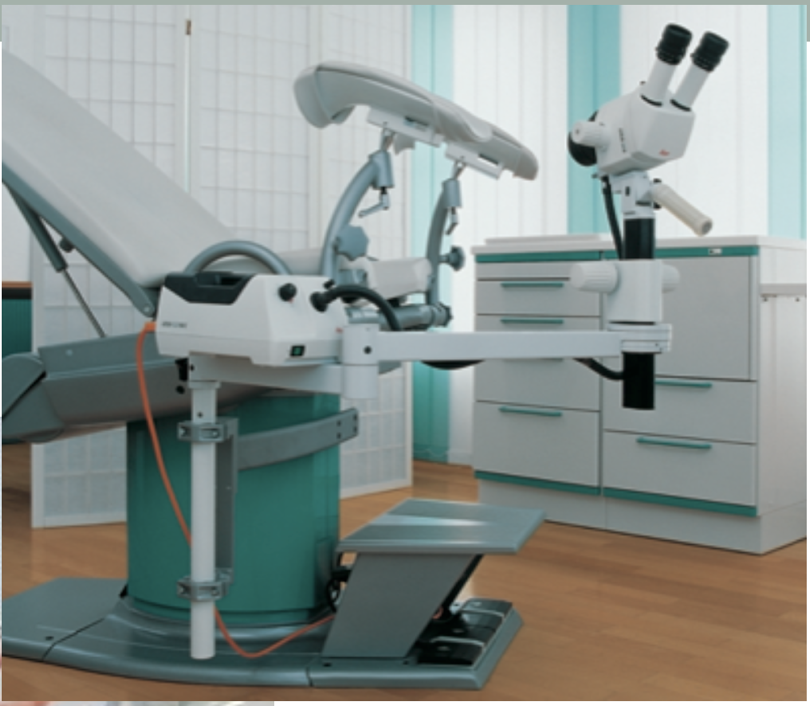


- Connaissiez-vous le principe de la colposcopie ?
  - Oui
  - Non
- En avez-vous déjà eu une ?
  - Oui
  - Non
  - Je ne m’en souviens pas

Les résultats des biopsies montrent dans certains cas des lésions qui sont dites « pré-cancéreuses ».

Dans ces cas-là, il faut retirer la partie atteinte du col de l’utérus.

Cela s’appelle une conisation.

La conisation est une opération qui se déroule à l’hôpital, le plus souvent sous anesthésie locale, c’est-à-dire qu’on injecte un produit dans le col de l’utérus qui supprime la douleur.

Cette conisation peut se faire en ambulatoire, c’est-à-dire sur une seule journée à l’hôpital.

Elle est réalisée en position gynécologique, sous colposcopie pour être la plus précise possible par un ou une gynécologue, au bistouri électrique (anse diathermique comme sur le schéma).

Le fragment de col retiré est ensuite envoyé en analyse.


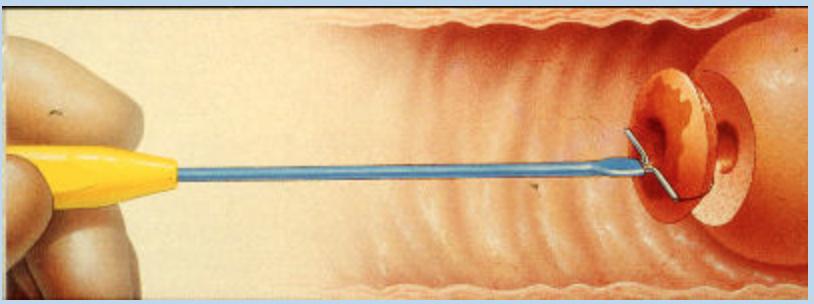


Schéma d’une conisation à l’anse diathermique

La conisation est un geste rapide, non douloureux après anesthésie.

Après l’opération, votre quotidien est le même.

La seule complication (rare) peut être des saignements abondants 10 jours après. Il faut signaler ces saignements à l’équipe médicale pour être examinée.

- Connaissiez-vous cette opération et ses principes ?
  - Oui
  - Non
- Si une conisation était indiquée dans votre cas, ces explications vous semblent-elles suffisantes ?
  - Oui
  - Non
  - Je ne m’en rends pas compte
- Ces explications vous semblent-elles rassurantes ?
  - Oui
  - Non
  - Je ne sais pas

Quand on est en détention, le moyen d’accès à l’hôpital se fait par une unité spécifique, l’UHSI.

- Connaissez-vous ce service ?
  - Oui
  - Non
- Y avez-vous déjà été hospitalisée ?
  - Oui
  - Non
- Si Oui : avez-vous un souvenir négatif de votre hospitalisation ?
  - Oui
  - Non
- Si Non : avez-vous une image négative de ce service ?
  - Oui
  - Non
  - Je ne me suis jamais posé la question
- Pensez-vous qu’être hospitalisée à l’UHSI vous prive de certaines activités que vous avez habituellement en détention ?
  - Oui
  - Non
  - Je ne sais pas
- Est-ce un frein si vous deviez aller dans ce service ?
  - Oui
  - Non
  - Je ne sais pas
- L’incertitude sur votre jour exact d’hospitalisation (comme c’est la règle) est-elle un frein pour aller à l’UHSI ?
  - Oui
  - Non
  - Je ne sais pas
- Le fait d’avoir une opération en ambulatoire (comme une conisation) vous paraît-il plus acceptable comme mode d’hospitalisation ?
  - Oui
  - Non
  - Je ne sais pas
